# Reliable Differentiation of a Bivalent Live *Salmonella* Vaccine and Field Strains: Multi-Supplier Validation of a Disc Diffusion Method

**DOI:** 10.3390/vetsci13030303

**Published:** 2026-03-23

**Authors:** Benjamin Bertin, Marie-Hélène Bayon-Auboyer, Mustapha Fellag, Véronique Piot, Sandra Debrouver, Anne-Christine Dufay-Lefort, Marc Henninger, Kévin Hervouet, Doris Mueller-Doblies

**Affiliations:** 1 Eurofins Cœur de France, 03017 Moulins, France; benjamin.bertin@ftfr.eurofins.com (B.B.);; 2 Labocea, 22440 Ploufragan, France; 3 Elanco France, Crisco Uno, Bâtiment C, 92317 Sèvres, France; 4 Elanco Austria GmbH, Quartier Belvedere Central, 1100 Vienna, Austria

**Keywords:** *Salmonella*, live vaccine, antimicrobial resistance pattern, disc diffusion assay

## Abstract

Zoonotic *Salmonella* is one of the most important foodborne zoonoses worldwide, with *Salmonella* Enteritidis (SE) and *Salmonella* Typhimurium (ST) being the most important serovars, which may be transmitted via poultry meat and eggs. Vaccination of breeding- and laying-hen flocks using live vaccines administered via drinking water has long been a well-recognized tool and contributed significantly to decreasing cases of salmonellosis in humans and its prevalence in the chicken sector. However, if a vaccine strain is detected in feces from recently vaccinated birds, it is imperative to have methods for reliably distinguishing between field and vaccine strains. In this study, we validated a disc diffusion method based on the antimicrobial resistance patterns of the vaccine strains contained in a live bivalent vaccine conferring protection against SE and ST. Discs from eight suppliers were sourced and tested for their suitability for reliably distinguishing between field and vaccine strains, a requirement fulfilled by discs from four suppliers. This method serves as an alternative approach for laboratories equipped to perform disc diffusion assays, and it is also a more cost-effective solution than real-time PCR assays.

## Simple Summary

Zoonotic *Salmonella*, particularly *Salmonella* Enteritidis (SE) and *Salmonella* Typhimurium (ST), represents a major cause of foodborne infections associated with poultry and eggs. Vaccination of breeding flocks and laying hens using live vaccines administered via drinking water has significantly reduced human salmonellosis and prevalence in the poultry sector. However, the detection of vaccine strains in feces from recently vaccinated birds requires reliable methods for distinguishing them from field strains. This study validates a disc diffusion method based on the antimicrobial resistance profiles of vaccine strains from a live bivalent vaccine against SE and ST. Discs from eight suppliers were evaluated, of which those from four proved suitable. This approach constitutes a simple and cost-effective alternative to real-time PCR for appropriately equipped laboratories.

## 1. Introduction

Zoonotic *Salmonella* strains originating from poultry pose a significant threat to human health, with salmonellosis being among the most important foodborne zoonoses worldwide [1]. Although a large variety of *Salmonella* strains can cause human foodborne illness, *Salmonella* Enteritidis (SE) and *Salmonella* Typhimurium (ST) are the most important zoonotic serovars that can be transmitted via poultry meat and eggs [2]. However, outbreaks are also often linked to foods other than poultry, such as food of non-animal origin, including fruits, vegetables, spices, and chocolate, to name a few [3,4,5,6,7]. It is therefore important for poultry flocks to be protected against both of these serovars through vaccination, among other measures.

Vaccination of breeding herds and laying hens has been a well-recognized tool since the 1990s and contributed significantly to decreasing cases of salmonellosis in humans and its prevalence in the chicken sector [8,9], and live vaccines have been shown to have several advantages over inactivated vaccines [10].

The bivalent vaccine AviPro™ SALMONELLA DUO contains two live attenuated vaccine strains: Sm24/Rif12/Ssq (a live attenuated *S*. Enteritidis strain) and Nal2/Rif9/Rtt (a live attenuated *S*. Typhimurium strain). These two strains provide homologous protection against both SE and ST and are excreted in the feces of vaccinated birds for a brief period after vaccination (usually for a few days) [11]. Accordingly, collecting fecal samples (pooled feces, boot swabs, and cloacal swabs) from recently vaccinated birds allows the detection of the vaccine strains.

It is therefore important to have a reliable method for distinguishing live *Salmonella* vaccines from field strains, and European legislation stipulates that such a method must be available for all live vaccines [12]. According to Regulation (EC) no. 2160/2003 [13], the culling of breeding flocks infected with *S*. Enteritidis or *S*. Typhimurium is mandatory. Laying-hen flocks infected with one of the two above-mentioned serovars do not have to be culled, but their eggs cannot be marketed as fresh table eggs, thus significantly reducing profitability for the producer. Therefore, in order to protect vaccinated flocks from unnecessary culling, it is necessary to ensure that vaccinated flocks can be reliably distinguished from flocks infected with a field strain [13]. For the AviPro™ vaccine strains, this requirement is fulfilled through the ability to distinguish vaccine strains from field strains using their specific antimicrobial resistance (AMR) patterns [14,15].

In the European Union and many other countries, zoonotic *Salmonella* strains are isolated from poultry flocks according to the procedure laid out in ISO 6579-1:2017 [16]. According to ISO 6579-1:2017, samples from the primary production stage must be tested using modified semisolid Rappaport Vassiliadis agar (MSRV) plates, which have proven to be more sensitive than liquid Rappaport Vassiliadis (RV) broth in detecting zoonotic *Salmonella* from poultry feces [17]. In regard to poultry fecal samples, MSRV agar is generally more sensitive and accurate than RV broth for detecting motile *Salmonella*, particularly at low contamination levels and in the presence of heavy competing flora. This is why the EU CRL and ISO 6579 method moved from a broth-only approach to an MSRV-based method for examining animal feces [18,19,20,21,22].

In practice, AviPro™ vaccine strains can hardly ever be cultured from samples from the primary production stage when they are tested solely following the ISO 6579-1:2017 method, as neither vaccine strain grows on MSRV plates due to the impaired motility [23]. Therefore, it is highly unlikely that AviPro™ live vaccines will be detected in monitoring samples from poultry flocks tested according to ISO 6579-1:2017.

However, if additional selective enrichment media, such as Mueller–Kauffmann Tetrathionate broth (MKTTn), are used, the chances of isolating vaccine strains from recently vaccinated birds are higher. This is the case in France, where the NFU-47-100 method, which prescribes the additional use of MKTTn broth, is used [24]. Therefore, and because vaccination of laying hen flocks using live vaccines has only been introduced recently in France, it has become necessary to provide French laboratories with a suitable and practical choice for differentiating field from vaccine strains.

Both vaccine strains are naturally occurring mutants, which were chosen following a lengthy selection process, resulting in strains with three independent selection markers each. One of these markers confers resistance to rifampicin, which is present in both vaccine strains. In addition, the *S*. Enteritidis vaccine strain Sm24/Rif12/Ssq carries a mutation conferring resistance to streptomycin, while the *S*. Typhimurium vaccine strain Nal2/Rif9/Rtt carries a mutation conferring resistance to nalidixic acid. These two markers are used to distinguish between the two vaccine strains, and this information may be relevant when birds are vaccinated with AviPro™ SALMONELLA DUO. Both vaccine strains are sensitive to erythromycin because of increased permeability of the cell membrane, while *Salmonella* field strains are intrinsically resistant to erythromycin. Therefore, sensitivity to erythromycin is an important feature of both vaccine strains and can be used to differentiate them from *Salmonella* field strains [14,15].

Traditionally, the specific AMR profile is used by adding the relevant antimicrobials to agar plates and assessing the growth or inhibition of growth of the colony to be tested after overnight incubation. However, an alternative method was sought for laboratories that do not prepare homemade agar plates or in situations where the relevant antibiotics may be difficult to source.

In 2021, a Luminex-based assay that targets seven specific single-nucleotide polymorphisms to differentiate the *S*. Typhimurium vaccine strain from wild-type strains was described [25]. This molecular test can distinguish *S.* Typhimurium field strains from the vaccine strain with 100% sensitivity and specificity within one working day. However, as a Luminex-based assay requires specialized laboratory equipment, it is unlikely to be a viable alternative for smaller and private laboratories. In 2024, a multiplex real-time PCR approach was validated and published. It offers an alternative for laboratories that prefer PCR diagnostics [26]. However, this PCR method is not currently available as a commercial kit; therefore, it needs to be established and validated locally, which may be a barrier in some cases.

To offer an additional, easy-to-use method for differentiating between field and vaccine strains, a disc diffusion method was developed and validated based on the French standard NF U47-107 [24], comparing the suitability and performance of antimicrobial discs from eight different suppliers. This method will allow some flexibility in terms of sourcing of consumables and offer an alternative approach to differentiating field strains from vaccines strains in any laboratory setting.

## 2. Materials and Methods

The live attenuated bivalent vaccine AviPro^TM^ SALMONELLA DUO (Elanco Deutschland GmbH, Bad Homburg, Germany) was used throughout the trial. The vaccine contains at least 1 × 10^8^ colony-forming units (CFUs) of a live attenuated *Salmonella* Enteritidis strain (SE, strain Sm24/Rif12/Ssq) and at least 1 × 10^8^ CFUs of a live attenuated *Salmonella* Typhimurium strain (ST, strain Nal2/Rif9/Rtt) per dose. In addition, the vaccine contains additives such as soy peptone, sucrose, gelatine, and HEPES buffer.

Field strains were collected from different regions in France over a 6-year period (2019–2025). The majority of field strains originated from boot swabs or hand swabs from *Gallus gallus*; however, a small number of isolates originated from flocks of the species *Meleagris gallopavo*. The isolates originated from different flocks, i.e., the same flocks, were not represented twice.

From a pure culture (the vaccine strain or field strain to be tested) obtained after 18 to 24 h on non-selective agar medium, we prepared a suspension in physiological saline solution adjusted to 0.5 (10^8^ CFUs/mL) according to the McFarland standard using a densitometer. From this suspension, a 1/10 dilution in physiological saline solution was prepared in order to achieve semi-confluent growth. Mueller–Hinton agar plates (BIORAD Mueller-Hinton/Agar, 120 mm square plates, #3563901, Bio-Rad, Marnes-la-Coquette, France) were placed at room temperature for 15 min before seeding [24].

A sterile swab was immersed in the 1/10 bacterial suspension (10^7^ CFU/mL) and used to inoculate the Mueller–Hinton agar plate within 15 min of inoculum preparation. Discs were then applied to the surface of the seeded plates using sterile forceps within 15 min of seeding [24].

As the aim was to provide a robust and flexible method that can be used in various regions, antimicrobial susceptibility discs from eight different suppliers were tested, allowing customers to use whichever discs are available in their country without having to validate the test via extrapolating from a different product. The discs used are listed in Table 1.

The tests were conducted by two French scientific laboratories.

**Table 1:** List of suppliers of the antimicrobial discs evaluated, including catalogue numbers. The discs from one supplier (Roth) did not produce reproducible results and are therefore not included in this list.

	Erythromycin	Streptomycin	Nalidixic Acid	Rifampicin
Biorad	66448 15 µg (4 × 50)	67418 10 µg (4 × 50)	68618 30 µg (4 × 50)	66648 5 µg (4 × 50)
MAST (MAST Diagnostic, Amiens, France)	E15C 15 µg (5 × 50)	S10C 10 µg (5 × 50)	NA30C 30 µg (5 × 50)	RP30C 30 µg (5 × 50)
Dutscher (Condalab) (Dutscher, Bernolsheim, France)	778152 15 µg (250)	778329 10 µg (250)	778245 30 µg (250)	778313 5 µg (250)
Fischer Oxoid (Fisher Scientific SAS, Illkirhc, France)	CT0020B 15 µg (5 × 50)	CT0047B 10 µg (5 × 50)	CT0031B 30 µg (5 × 50)	CT0104B 30 µg (5 × 50)
Liophilchem (Liophilchem, Roseto degli Abruzzi, Italy)	9024 15 µg (5 × 50)	9040 10 µg (5 × 50)	9001 30 µg (5 × 50)	9039 30 µg (5 × 50)
Becton Dickinson (Becton Dickinson France, Le Pont doe Claix, France)	230793 15 µg	230942 10 µg	230874 30 µg	231544 5 µg
I2A (I2A Diagnostics, Montpellier, France)	#065183 15 µg (5 × 50)	#065090 10 µg (5 × 50)	#065002 30 µg (5 × 50)	#065085 30 µg (5 × 50)

After the antimicrobial discs were applied, the plates were incubated at 37 ± 2 °C for 18 to 24 h, with incubation starting within 15 min of the discs being placed. A reading was only validated if two conditions were met: there was proper confluence of colonies, and the culture was pure [24]. Inhibition diameters were measured using a caliper or an automated reading system such as SIRSCAN (Axonlab, Baden-Daettwill, Switzerland), and diameters were expressed in mm.

A repeatability study was performed to assess inter-supplier variability with respect to the antibiotics for the vaccinal strains and some reference strains of *S*. Enteritidis and *S*. Typhimurium. Erythromycin and rifampicin discs were tested for the two serotypes, and streptomycin and nalidixic acid discs were tested for *S*. Enteritidis and *S*. Typhimurium, respectively.

Discriminatory criteria were standardized in two steps: First, vaccine strains (vaccine isolates from water or fecal samples, collected as part of vaccination or routine quality controls) and wild strains were subjected to an interlaboratory test conducted on 10 strains, enabling a comparison of values on a set of common strains. As a second step, a reproducibility analysis was carried out on 30 vaccinal strains and 60 field strains for each serotype. Statistical tests (using coefficients of variation) were performed to determine the diameter intervals, allowing the categorization of wild-type and vaccine strains. All statistical analyses were performed using GraphPad Prism software (version 10.6.1).

## 3. Results

Table 2 shows the inhibition zones that were obtained with the discs from the eight suppliers.

For the discs containing erythromycin, streptomycin, and nalidixic acid, the discs from seven suppliers allowed for clear differentiation between the field and vaccine strains.

For erythromycin (15 µg discs), streptomycin (10 µg discs), and nalidixic acid (30 µg discs), discs from the following providers were able to distinguish between field and vaccine strains: Biorad, Mast, Dutscher (Condalab), Fisher Oxoid, Liophilchem, Becton Dickinson, and I2A.

For rifampicin, only the 30 µg discs could correctly distinguish between vaccine strains and field strains. Discs sourced from Mast, Fisher Oxoid, Liophilchem, and I2A, which provide 30 µg discs, allowed for this differentiation. The 5 µg rifampicin discs offered by three suppliers (BioRad, Dutscher, and Becton Dickinson) did not enable reliable differentiation and are therefore not recommended.

Following reproducibility analyses performed on the studied strains, no significant variation was identified among the different analytical groups evaluated.

**Figure 1:**
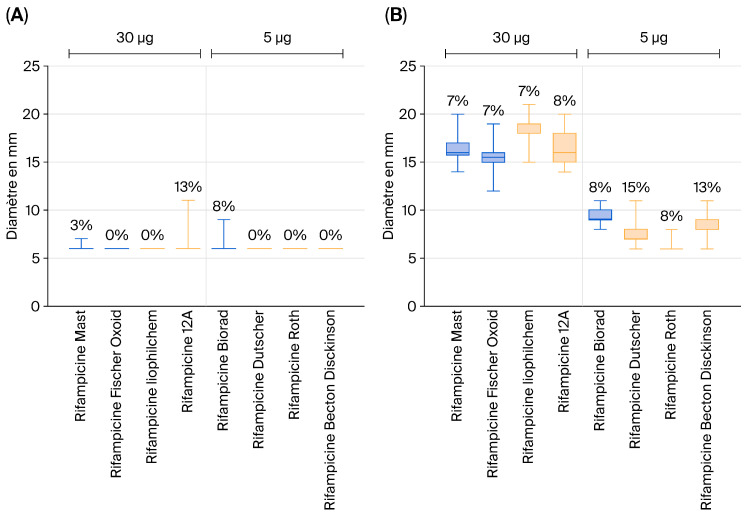
Inhibition zone diameters for vaccine strains of *Salmonella* Typhimurium (**A**) and *S*. Typhimurium field strains (**B**) obtained using rifampicin discs.

Specifically, for the 5 µg rifampicin discs, no significant differences were observed. As illustrated by the graphs derived from our raw data and shown in Figure 1, no notable differences in inhibition zone diameters were detected among the vaccine strains of *Salmonella* Typhimurium (Figure 1A) and the *S*. Typhimurium field strains (Figure 1B). The same observation was made for *Salmonella* Enteritidis .

This analysis was conducted on a total of 30 vaccine strains and 60 field (wild-type) strains.

These results suggest that 5 µg rifampicin discs cannot reliably discriminate between vaccine and field strains based on the observed inhibition zone diameters.

Generally, Roth discs did not demonstrate good repeatability of results and are therefore not recommended. The decision to exclude Roth is based on the analytical results obtained during the comparative study. For rifampicin and erythromycin, no significant differences were observed between the different suppliers, regardless of the method used. Although slight variations in inhibition zone diameters were noted, these differences were minor and did not compromise result interpretation. This inter-supplier consistency was also observed for streptomycin, for which inhibition zone diameters were identical (6 mm) across all eight suppliers independently of the method employed. However, an exception was identified for Roth. Significantly higher inhibition zone diameters (ranging from 23 mm to 30 mm) were observed in this study, and only for this supplier, even though all antibiotics were used at the same time.

This anomaly was noted exclusively for this supplier, thereby ruling out methodological bias or technical issues during the study.

These findings suggest that the observed discrepancy is not related to the analytical method or the strain tested; rather, it is a supplier-specific issue, possibly linked to a particular lot of discs.

Based on our analysis, the recommended cut-off values to be used when analyzing *Salmonella* isolates from poultry are summarized in Table 3.

## 4. Discussion

Live *Salmonella* vaccines are widely used in most European countries and elsewhere. They are mostly used in breeding flocks, but they are also applied in commercial laying-hen flocks. Vaccination of broiler herds is also becoming increasingly common in selected countries because of increasing prevalences seen in this production sector. Live vaccines offer several advantages over inactivated vaccines, such as the fact that they are easy to administer via drinking water, do not generate stress for the birds, and initiate a localized, humoral and cellular immune response in the gut [10]. However, as live vaccines are shed for a few days post vaccination and may be detected in some cases in fecal samples, it is imperative to offer a reliable method for differentiating between *Salmonella* field and vaccine strains. In the EU, where *S*. Enteritidis and *S*. Typhimurium are regulated serovars according to Regulation 2160/2003 [13], isolating either of these two serovars from flocks during routine monitoring will trigger certain consequences, depending on the production sector. In the case of breeding flocks, a confirmed case will result in the culling of the flocks, while the eggs from commercial laying-hen flocks testing positive for either SE or ST will be subjected to heat treatment [13]. In some countries, laying hen flocks even have to be culled. Therefore, there must be reliable tests that can correctly exclude or confirm the presence of a *Salmonella* field or vaccine strain.

Traditionally, differentiation is performed using non-selective agar plates containing either rifampicin, erythromycin, streptomycin, or nalidixic acid at concentrations set according to the SPC [27]. This method has been used successfully since the 1990s, when the two vaccines strains were first registered; however, in some instances, laboratories look for alternative methods that better suit their work routines. In particular, the need to prepare homemade agar plates containing the relevant antibiotics seems to be a hurdle for some laboratories. Furthermore, rifampicin is an expensive antibiotic that must be added to the agar plates at high concentrations and is not readily available in all regions.

Modern molecular alternatives for differentiating the two vaccine strains from field strains using a real-time PCR assay [26] or differentiating the *Salmonella* Typhimurium vaccine strain from field strains using a Luminex-based assay have been described [25] and are now established in some reference laboratories. However, both methods require certain laboratory equipment and molecular capabilities.

We therefore sought to validate a method that may be the ideal solution for laboratories that are well-equipped for basic bacteriology and culture but lack molecular capabilities. It is a cost-effective, simple, and reliable bridge between the old “home-made plate” method and the high-tech molecular methods.

In this study, we established criteria for distinguishing vaccine strains of the AviPro™ SALMONELLA DUO vaccine from wild-type *Salmonella* Enteritidis and *Salmonella* Typhimurium strains using a disc diffusion assay. It may be a popular alternative to the abovementioned methods, particularly in laboratories where agar plates are bought as ready-to-use products, rendering them incapable of producing plates containing specific antibiotics.

The vaccine strains used throughout the study were grown from the commercially available vaccine rather than from droppings of vaccinated birds. Due to three separate and independent mutations, the risk of back-mutation of the vaccine strains is considered negligible [15], and their stability has been demonstrated through their worldwide use since the 1990s. We therefore would not expect differing results if vaccine strain isolates from the field were used instead.

The results show that erythromycin and streptomycin can effectively distinguish SE vaccine strains, while erythromycin and nalidixic acid are suitable for differentiating ST strains. Regardless of the supplier, discs impregnated with these antibiotics enable clear differentiation between vaccine and wild strains of SE and ST. However, the use of rifampicin proved more problematic: the 5 µg discs offered by some suppliers do not allow reliable differentiation. By contrast, the 30 µg discs supplied by Mast, Fisher Oxoid, Liophilchem, and I2A perform better and can be used as a complementary tool in the discrimination protocol.

## 5. Conclusions

This validated disc diffusion method now offers an additional, reliable, cost-effective, easy-to-perform methodology that is readily available in different regions around the world and does not require expensive laboratory equipment.

## Figures and Tables

**Table 2:** (a) Table summarizing inhibition diameters as a function of coefficients found in the context of categorizing the vaccine strain/field strain *S*. Enteritidis. Results are presented as [min, max]. (b) Table summarizing inhibition diameters as a function of coefficients found in the context of categorizing the vaccine strain/field strain *S*. Typhimurium. Results are presented as [min, max]. N.I.—no inhibition.

(a) *Salmonella* Enteritidis (Diameter in mm)						
	Erythromycin		Streptomycin		Rifampicin	
	Vaccine	Field	Vaccine	Field	Vaccine	Field
BioRad	[16, 19]	[6, 9]	N.I.	[20, 22]	N.I.	[8, 10]
Mast	[15, 18]	[6, 9]	N.I.	[20, 24]	N.I.	[14, 16]
Dutscher	[16, 20]	[6, 11]	N.I.	[19, 22]	N.I.	[6, 8]
Roth	[15, 20]	[7, 12]	N.I.	[23, 26]	N.I.	[6, 6]
Fisher Oxoid	[15, 18]	[6, 9]	N.I.	[19, 22]	N.I.	[13, 15]
Liophilchem	[14, 17]	[6, 8]	N.I.	[19, 22]	N.I.	[16, 18]
I2A	[19, 20]	[9, 12]	N.I.	[21, 24]	N.I.	[14, 16]
Becton Dickinson	[16, 19]	[6, 10]	N.I.	[20, 22]	N.I.	[6, 8]
(b) *Salmonella* Typhimurium (Diameter in mm)						
	Erythromycin		Nalidixc Acid		Rifampicin	
	Vaccine	Field	Vaccine	Field	Vaccine	Field
BioRad	[20, 23]	[7, 11]	[14, 16]	[23, 26]	N.I.	[8, 11]
Mast	[20, 23]	[7, 10]	[12, 15]	[22, 25]	N.I.	[14, 18]
Dutscher	[21, 24]	[7, 12]	[15, 18]	[25, 28]	N.I.	[6, 9]
Roth	[20, 23]	[8, 13]	[16, 18]	[25, 28]	N.I.	[6, 7]
Fisher Oxoid	[19, 23]	[7, 10]	[13, 16]	[24, 26]	N.I.	[14, 17]
Liophilchem	[19, 22]	[6, 9]	[16, 18]	[26, 28]	N.I.	[17, 20]
I2A	[23, 25]	[9, 13]	[15, 17]	[25, 28]	N.I.	[15, 18]
Becton Dickinson	[20, 23]	[7, 11]	[15, 17]	[24, 27]	N.I.	[7, 10]

**Table 3:** (a) Recommended cut-off values when assessing zones for the *S*. Enteritidis vaccine strain in comparison to field strains. (b) Recommended cut-off values when assessing zones for the *S*. Typhimurium vaccine strain relative to field strains.

(a) *Salmonella* Enteritidis (Diameter in mm)						
	Erythromycin		Streptomycin		Rifampicin	
	Vaccine	Field	Vaccine	Field	Vaccine	Field
BioRad	>15	<10	<7	>19	Not suitable	Not suitable
Mast	>14	<10	<7	>19	<7	>13
Dutscher	>15	<12	<7	>18	Not suitable	Not suitable
Fisher Oxoid	>14	<10	<7	>18	<7	>12
Liophilchem	>13	<9	<7	>18	<7	>15
I2A	>18	<13	<7	>20	<7	>13
Becton Dickinson	>15	<11	<7	>19	Not suitable	Not suitable
(b) *Salmonella* Typhimurium (Diameter in mm)						
	Erythromycin		Nalidixic Acid		Rifampicin	
	Vaccine	Field	Vaccine	Field	Vaccine	Field
BioRad	>19	<12	<17	>21	Not suitable	Not suitable
Mast	>19	<11	<16	>20	<7	>13
Dutscher	>20	<13	<19	>23	Not suitable	Not suitable
Fisher Oxoid	>18	<11	<17	>22	<7	>13
Liophilchem	>18	<10	<19	>25	<7	>16
I2A	>22	<14	<18	>24	<7	>14
Becton Dickinson	>19	<12	<18	>23	Not suitable	Not suitable

## Data Availability

The original contributions presented in this study are included in the article. Further inquiries can be directed to the corresponding author(s).
